# Predictors of community-based health insurance enrollment among reproductive-age women in Ethiopia based on the EDHS 2019 dataset: a study using SHAP analysis technique, 2024

**DOI:** 10.3389/fpubh.2025.1448055

**Published:** 2025-03-20

**Authors:** Sisay Yitayih Kassie, Solomon Abuhay Abebe, Mekdes Wondirad, Samrawit Fantaw Muket, Ayantu Melke, Alex Ayenew Chereka, Adamu Ambachew Shibabaw, Abiy Tasew Dubale, Yitayish Damtie, Habtamu Setegn Ngusie, Agmasie Damtew Walle

**Affiliations:** ^1^Department of Health Informatics, School of Public Health, College of Medicine and Health Science, Hawassa University, Hawassa, Ethiopia; ^2^School of Public Health, College of Medicine and Health Sciences, Hawassa University, Hawassa, Ethiopia; ^3^Department of Health Informatics, College of Health Science, Mattu University, Mattu, Ethiopia; ^4^Department of Public Health, College of Medicine and Health Science, Injibara University, Injibara, Ethiopia; ^5^Department of Health Informatics, School of Public Health, College of Medicine and Health Sciences, Woldia University, Woldia, Ethiopia; ^6^Department of Health Informatics, Institute of Public Health, Asrat College of Medicine and Health Science, Debrebirhan University, Deberebrihan, Ethiopia

**Keywords:** SHAP analysis, community-based health insurance, enrollment, reproductive-age women, Ethiopia

## Abstract

**Background:**

Out-of-pocket payments for health services can lead to health catastrophes and decreased service utilization. To address this issue, community-based health insurance has emerged as a strategy to provide financial protection against the costs of poor health. Despite the efforts made by the government of Ethiopia, enrollment rates have not reached the potential beneficiaries. Therefore, this study aimed to predict and identify the factors influencing community-based health insurance enrollment among reproductive-age women using SHapley Additive exPlanations (SHAP) analysis techniques.

**Method:**

The study was conducted using the recent Demographic Health Survey 2019 dataset. Eight machine learning algorithm classifiers were applied to a total weighted sample of 9,013 reproductive-age women and evaluated using performance metrics to predict community-based health insurance enrollment with Python 3.12.2 software, utilizing the Anaconda extension. Additionally, SHAP analysis was used to identify the key predictors of community-based health insurance enrollment and the disproportionate impact of certain variables on others.

**Result:**

The random forest was the most effective predictive model, achieving an accuracy of 91.64% and an area under the curve of 0.885. The SHAP analysis, based on this superior random forest model, indicated that residence, wealth, the age of the household head, the husband’s education level, media exposure, family size, and the number of children under five were the most influential factors affecting enrollment in community-based health insurance.

**Conclusion:**

This study highlights the significance of machine learning in predicting community-based health insurance enrollment and identifying the factors that hinder it. Residence, wealth status, and the age of the household head were identified as the primary predictors. The findings of this research indicate that sociodemographic, sociocultural, and economic factors should be considered when developing and implementing health policies aimed at increasing enrollment among reproductive-age women in Ethiopia, particularly in rural areas, as these factors significantly impact low enrollment levels.

## Background

The Sustainable Development Goals (SDG) emphasize ensuring that all people have access to quality healthcare services without financial hardship by 2030 (1). However, across the world, a million citizens have suffered catastrophic financial shocks due to direct payments for healthcare services (2), resulting in half of the population lacking access to basic healthcare services, 25 million households living in poverty, and 44 million households burdened with health expenses (3). Approximately 90% of these catastrophic events have been observed in Sub-Saharan African countries (4–6). In Ethiopia, the situation is exacerbated by low economic development, a weak health system, and rapid population growth.

To address this financial catastrophe, Community-Based Health Insurance (CBHI) is a key component of the Ethiopian government’s healthcare financing reform strategy. This strategy aims to promote equitable access to healthcare, increase financial protection against medical expenses, encourage cost-sharing between the government and citizens, and enhance domestic resource mobilization for the health sector while fostering social inclusion in healthcare (7, 8). The reform strategy was implemented from 2015/16 to 2019/20 with the goal of fostering mutual aid and solidarity (9, 10).

To ensure this, the government mobilizes the community and provides health education on the importance of enrolling in community health insurance. However, enrollment levels in the health insurance scheme have been unsatisfactory. Research findings revealed that 3% of Nigeria, 4.7% of Ethiopia, 7% of Kenya, and 7.56% of East Africa reproductive-age women enrolled in a health insurance scheme (11–15). The progress of CBHI enrollment in Ethiopia is low, despite the program being launched to achieve 80% coverage in all districts and reach 80% of its population by 2020, with the aim of reducing out-of-pocket medical expenses that affect 85% of its population (16, 17).

In the pilot year, CBHI enrollment was extremely impressive, rising to 41% in the first year and 48% in the second year. The program has experienced consistent growth in enrollment over the past 2 years, although there is a significant 18% churn rate among households that participated in the first year and subsequently stopped making payments in the second year (18). Consequently, scholars aim to investigate the level of CBHI enrollment and the factors hindering it (13, 19, 20). For instance, previous studies assess willingness, its impact on quality of life, levels of satisfaction, coverage, and enrollment status in the program schemas (13, 21–26). Additionally, studies have explored factors influencing enrollment status, such as media exposure, women’s educational status, marital status, place of residence, head of household’s sex, wealth status, age of household heads, husband’s educational background, religion, and region (12, 15, 21, 25).

In addition, previous researchers examined community-based health insurance enrollment and geographical variations using the Ethiopian Demographic and Health Survey (EDHS) datasets from 2016 and 2019. A recent study utilizing the EDHS 2019 dataset focused on assessing community-based health insurance enrollment among reproductive-age women, as they and their children greatly benefit from this health insurance system without facing financial catastrophe (13, 25, 27). However, this study employed classical statistical models. Therefore, this research aims to uncover new insights into the factors influencing community-based health insurance enrollment among reproductive-age women by using a nonlinear machine learning model. To achieve this objective, we applied SHapley Additive exPlanations (SHAP) analysis to identify significant features and examine the disproportionate effects of certain variables using the most recent nationally representative EDHS 2019 dataset (28–32). Policymakers, health planners, and program managers working in health insurance initiatives aimed at achieving universal health coverage (UHC) and improving the financial security of reproductive-age women can use these findings to make evidence-based decision-making.

## Method

### Study area, period, and design

This study was conducted using secondary data from the 2019 Ethiopia Demographic and Health Survey (EDHS). A cross-sectional study design was employed. Ethiopia consists of 12 regional states and two administrative cities, Addis Ababa and Dire Dawa. Furthermore, it is subdivided into various administrative zones and woredas. Located in the Horn of Africa, Ethiopia is bordered by Eritrea to the north, Djibouti and Somalia to the east, Sudan and South Sudan to the west, and Kenya to the south. The Ethiopian Public Health Institute and the Central Statistical Agency collaborated to gather the 2019 EDHS data, which was collected between March 21 and June 28, 2019. The purpose of this data collection was to provide information for tracking the progress of health sector targets outlined in the Growth and Transformation Plan.

### Source and study population

All reproductive-age women (15–49), whether permanent residents of the selected households or guests who stayed there the night before the survey, constituted the source population. In contrast, all reproductive-age women who completed the survey formed the study population.

### Sample size

A total of 9,013 reproductive-age women participated in this study.

### Study variables

#### Dependent variable

The dependent variable for this study is the enrollment of reproductive-age women in community-based health insurance, which is categorized as “yes” and “no.” Furthermore, the question posed was, “Have you ever enrolled in community-based health insurance?” The response was “yes” if the women had been members of community-based health insurance and “no” if they had not enrolled in it.

#### Independent variable

The predictor features for community-based health insurance enrollment include the sex of the household head, residence, region, women’s age, education level, wealth status, husband’s educational level, age of the household head, number of children under 5 years of age, and media exposure, which are expected predictors of community-based health insurance enrollment among reproductive-age women.

### Data source

The dataset for this study is available on the Measure of EDHS program and can be accessed from the^1^ website after a clear explanation of the study’s objective.

### Data processing and statistical analysis

STATA version 14 software was used to extract datasets from the data source, manage and prepare data, and recode variables to achieve the desired classification for further analysis. To ensure the representativeness of survey results at the national level, sampling weights were applied during the analysis. Python version 3.11 software, using the Anaconda Jupyter Notebook editor, was used for data collection, preprocessing, model training, testing, and predicting CBHI enrollment among reproductive-age women.

In this study, SHAP analysis of feature importance was used to evaluate the relationship between the predictors and the outcome variable, thereby identifying the most significant predictor of CBHI enrollment. To accomplish this, seven steps were undertaken in supervised machine learning: data collection, data preparation, model selection, model training, model evaluation, parameter tuning, and prediction.

### Data preparation, preprocessing, and feature selection

This study involved data cleaning, feature engineering, and data splitting to detect and remove outliers, manage noisy data, address inconsistencies, handle missing values, and correct imbalances in class variables. To address outliers in the dataset, we employed Winsorizing, which is preferred over trimming when the number of outliers is small and the range of values is not unrealistically extreme. To resolve the class imbalance in the outcome variable, we utilized various data balancing techniques to prevent biased predictions of the class variable. Consequently, we applied the Synthetic Minority Oversampling Technique (SMOTE) to balance the dataset (29, 33–35).

Furthermore, the entire dataset is divided into training and testing sets by randomly allocating 80% of the dataset for model training and 20% for testing the machine learning (ML) model using 10-fold cross-validation. Finally, SHAP analysis was utilized in this study for feature selection (28, 36) due to its consistency and unbiased explainability, which provides an intuitive visualization of model predictions that allows users to easily understand the contribution of each feature to the final prediction. Although SHAP analysis provides both local and global interpretability, this study focused on global interpretability. However, as it provides insights into the overall behavior of the model. In addition, waterfall plot analysis was employed to observe the impact of predictive features on the positive predictive class “yes” for enrollment in CBHI. A waterfall plot provides a detailed breakdown of how each feature contributes to the final prediction for a specific instance. Each bar in the plot represents a feature, and the length of the bar indicates the magnitude and direction of its contribution.

Positive SHAP values, shown by the bar to the right of the baseline, indicate features that have a positive effect on the prediction. Higher values of these features lead to higher predictions, while lower values result in lower predictions. Conversely, negative SHAP values, shown by the bar to the left of the baseline, indicate features that have a negative effect on the prediction. Higher values of these features contribute to lower predictions, while lower values yield higher predictions. Additionally, analyzing the SHAP waterfall plot provides insights into the relative importance and directionality of various features in determining the classification outcome for a specific sample.

### Model selection training and evaluation

We used eight machine learning algorithms: linear logistic regression, Random Forest (RF), Decision Tree (DT), support vector machine (SVM), Gaussian Naive Bayes (GNB), K-nearest neighbors (KNN), XGBoost (XGB), and Light Gradient Boosting (LGB). These algorithms accurately and precisely predict community-based health insurance enrollment. They were chosen based on the target variable, as our class variable is binary, classified as “yes” if women are enrolled in CBHI and “no” if they are not. In this study, we implemented a straightforward strategy to divide the total dataset into two parts: a training dataset comprising 80% of the total and a testing dataset representing the remaining 20%.

The classification of the dataset into 80 and 20% was conducted after careful consideration and experimentation regarding the dataset’s number of features, types of problems, strength of noise or outliers, interpretability, computational efficiency, sample size, the types of machine learning algorithms employed, and their complexity levels. After model selection and classification, the classifiers were trained on both unbalanced and balanced datasets, and their performance was evaluated using the 80% training dataset with 10-fold cross-validation.

The performance of each algorithm was compared using the confusion matrix (37). The accuracy of actual and predicted classes of community-based health insurance enrollment has been visualized through the confusion matrix, which includes false positive (FP), true positive (TP), true negative (TN), and false negative (FN) (38). However, accuracy and the receiver operating characteristic (ROC) curve were utilized for model evaluation and visualization.

The ROC curve works based on probability, the area under the curve (AUC) that tells how much the model is capable of distinguishing between classes. Therefore, the higher the AUC, the better the model is at predicting true classes as “true” and false classes as “false” (39). After the comparison, the best-performing predictive model was selected and fine-tuned with hyperparameters for final predictions on the unseen test dataset reserved for this purpose.

### Hyperparametric tuning

Hyperparameter tuning is the process of finding the optimal hyperparameters for a machine-learning model. Hyperparameters are settings that are not learned during the training process but are determined before training begins. Tuning these hyperparameters is essential for achieving the best possible model performance. Accordingly, the Optuna hyperparameter tuning framework has been used in this study due to its popularity and the various advantages of optimization compared to grid searching and randomized hyperparameter tuning.

## Results

### Sociodemographic characteristics of study participants

Among a total of 9,013 reproductive-age women, 6,014 (66.7%) of the study participants were rural residents. Regarding the sex of the household head, 6,463 (71.71%) were male. Many of the participants were from the Oromia region, with 1,070 (11.9%), followed closely by South Nation, Nationalities, and Peoples (SNNP) at 1,028 (11.4%). Most participants, around 6,638 (73.65%), had one or no children under 5 years old. Additionally, approximately 5,051 (56.04%) had no media exposure, and 39.30% of their husbands had no education, followed by those with primary education (Table 1).

**Table 1:** Sociodemographic characteristics of reproductive-age women in Ethiopia using the 2019 DHS dataset, 2024.

Variables	Categories	Frequency (weighted)	Percentage (%)
CBHI enrollment status	Yes	1,830	20.3
	No	7,183	79.7
Residence	Urban	2,999	33.3
	Rural	6,014	66.7
Region	Tigray	743	8.2
	Afar	642	7.1
	Amhara	964	10.7
	Oromia	1,069	11.9
	Somali	648	7.1
	Benishangul-Gumuz	750	8.3
	SNNP	1,028	11.4
	Gambela	750	8.3
	Harari	768	8.5
	Addis Ababa	826	9.4
	Dire Dawa	825	9.1
Sex_hh_head	Male	6,463	71.71
	Female	2,550	28.29
Family size	≤5	4,662	51.73
	>5	4,351	48.27
No_under_5_ child	≤1	6,638	73.65
	>1	2,375	26.35
Age_hh_head	≤24	596	6.61
	25–39	3,533	39.20
	≥40	4,884	54.19
Wealth	Poorest	2,055	22.80
	Poorer	1,357	15.06
	Middle	1,292	14.33
	Richer	1,367	15.17
	Richest	2,942	32.64
Media exposure	No media exposure	5,051	56.04
	Has media exposure	3,962	43.96
Husband educ_level	No education	3,542	39.30
	Primary	3,531	39.18
	Secondary	1,171	12.99
	Higher	769	8.53

### Analysis of machine learning algorithms for community-based health insurance enrollment

#### Balancing dataset

**Figure 1:**
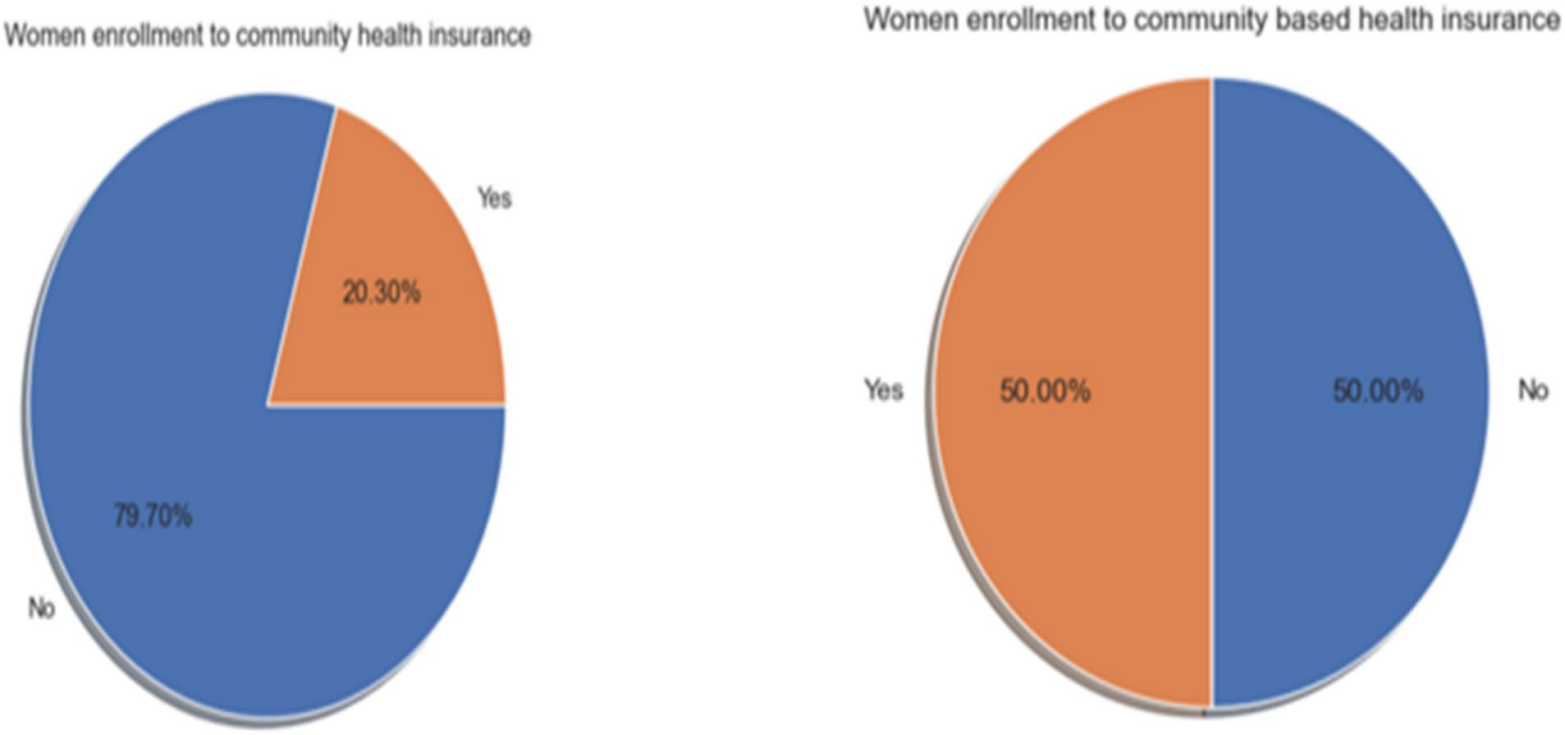
Distribution of CBHI enrollment among reproductive-age women before and after SMOTE, using EDHS 2019 data in Ethiopia (2024).

In this study, the synthetic minority over-sampling technique (SMOTE) was employed to address the issue of imbalanced data in the dataset. As a result, the SMOTE oversampling technique generates 5,353 additional minority class variables from the outcome class variables (Figure 1).

### Model development and performance evaluation

**Figure 2:**
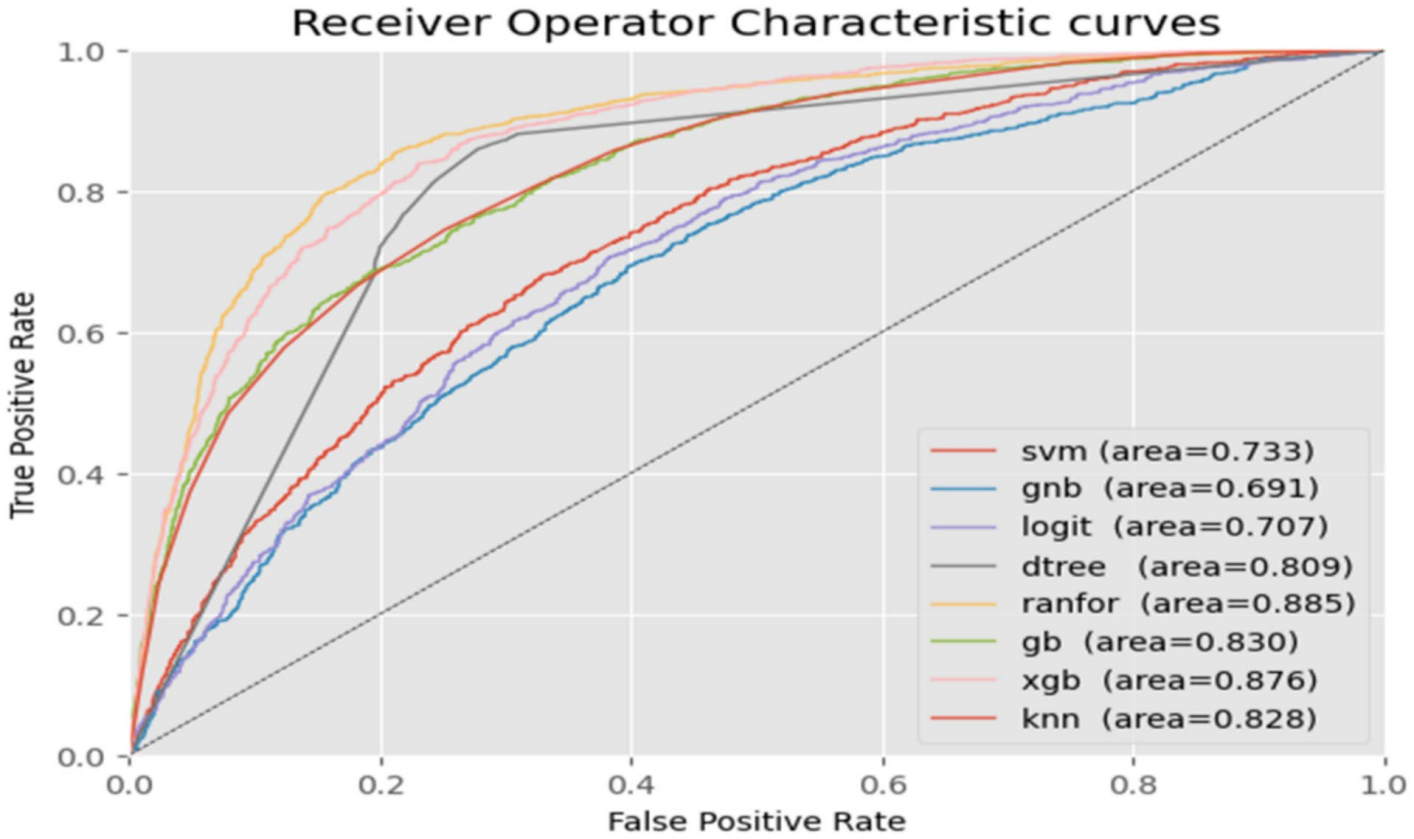
A comparison of the performance of machine learning algorithms using the area under the curve (AUC-ROC) based on the EDHS 2019 data from Ethiopia, 2024.

After the model selection predicting CBHI enrollment was conducted to assess the accuracy of predicting positive class instances as positive and negative class instances as negative. Following a thorough evaluation using performance metrics, the random forest machine learning algorithm was identified as the top predictor for CBHI enrollment among reproductive-age women in Ethiopia, achieving an accuracy of 82.5% and an AUC of 0.880, followed by the XGBoost machine learning algorithm, which achieved an accuracy of 80.0% and an AUC of 0.853. This result may be biased due to the unbalanced class variable. Consequently, the training dataset was balanced using SMOTE oversampling techniques, and unexpectedly, random forest emerged as the top-performing machine learning algorithm, achieving an accuracy of 91.32% and an AUC of 0.885. The overall performance comparison of machine learning algorithms for community-based health insurance enrollment is presented in Figure 2.

### Hyperparameter tuning with random forest

Scikit-learn provides a set of sensible default hyperparameters for all models, including random forests. However, these defaults are not guaranteed to be optimal for every problem. To maximize the performance of the random forest, several hyperparameters were optimized through one hundred trials within a specified search space. These include the number of decision trees in the forest (number of estimators), the number of features considered by each tree when splitting a node (maximum features), the minimum number of samples required to split an internal node (minimum samples split), the minimum number of samples required to be at a leaf node (minimum samples leaf), and the number of samples drawn from independent variables to train each tree (maximum samples). The optimization process used an 80/20 split method, allocating 80% of the data for model training and the remaining 20% for testing the model. The default hyperparameters set by Scikit-learn and our optimized hyperparameters are shown in Table 2.

**Table 2:** The default and optimally tuned hyperparameters of the Random Forest model for reproductive-age women in Ethiopia, 2024.

Hyperparametric tuning	Default	Optimal values
No of trees	100	175
No of features need to be considered for the best split	The square root of the number of features	2.83
Mini no of sample is required to split internal nodes	2	5
Mini no of samples required to be at the leaf of the nodes	1	1
Max no of samples to draw from X to train each estimator	None	0.85

### Feature selection

**Figure 3:**
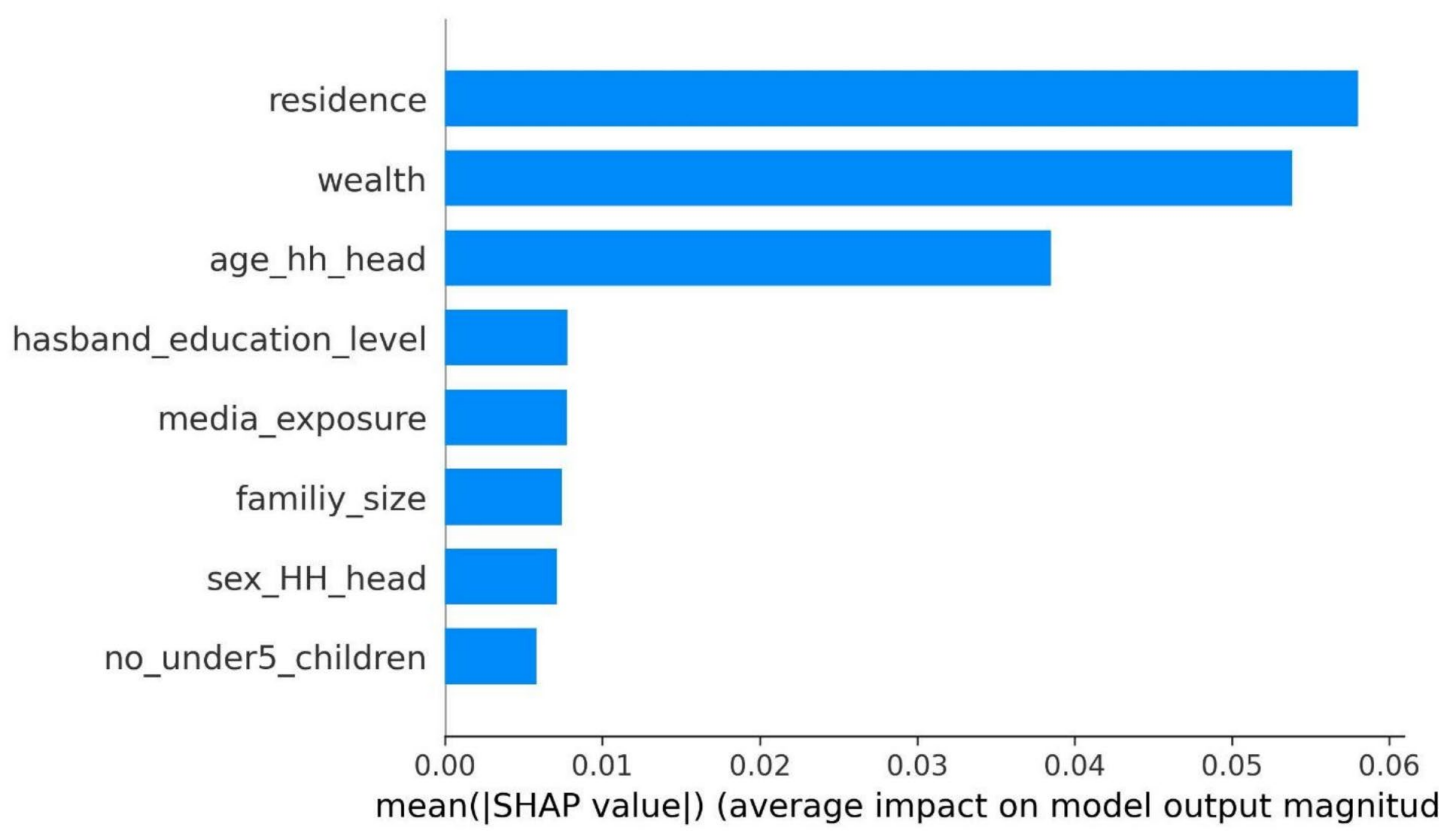
A SHAP global analysis feature importance plot with the most outfitted random forest model using the EDHS 2019 dataset, Ethiopia, 2024.

The SHAP global analysis, using the best-performing machine learning algorithm, was conducted to identify the most important feature predictors of CBHI enrollment among reproductive-age women. This analysis utilizes the mean absolute value for each predictor across the entire dataset, quantifying the feature’s contribution to predict CBHI enrollment among reproductive-age women. Characteristics with higher mean absolute SHAP values hold greater influence, and the predictors are arranged in descending order based on their impact on the outcome variable. The analysis revealed that residence, wealth, the age of the household head, media exposure, the husband’s educational level, the number of children under five, the sex of the household head, and family size were the most significant features predicting CBHI enrollment among reproductive-age women. According to this study, place of residence, wealth status, and age of the household head are the most influential features predicting the outcome variable in the model (Figure 3).

### SHapley Additive exPlanations model evaluation and explanation

**Figure 4:**
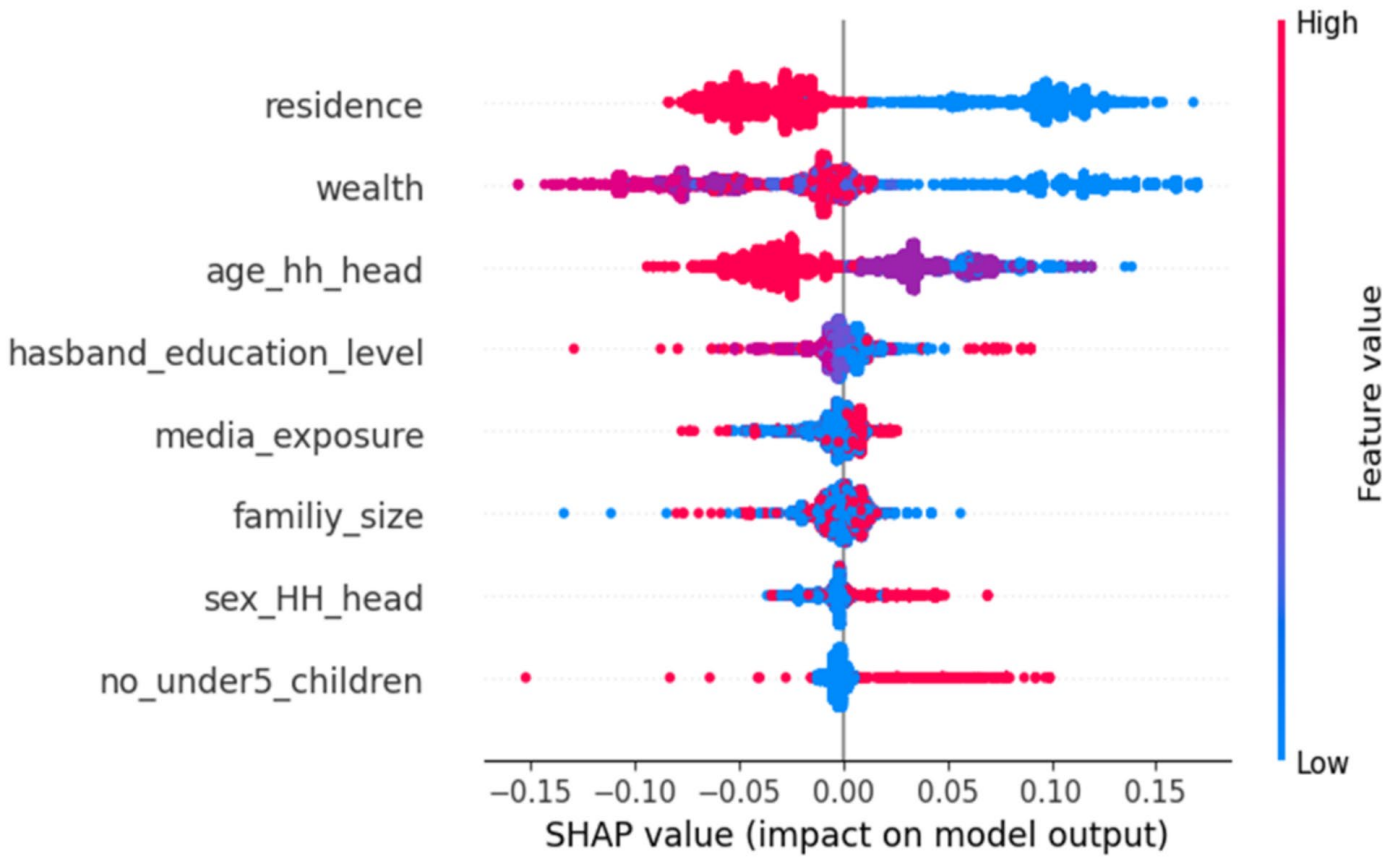
A SHAP analysis of the feature importance plot, ranked by the mean absolute SHAP values generated by the random forest model of CBHI enrollment for reproductive-age women in Ethiopia, 2024, using the 2019 EDHS dataset.

The beeswarm plot was used to identify how each feature impacts the model’s predictive performance across all datasets. The usefulness of the input features in predicting a target class is represented by feature importance. To achieve this, the feature importance technique provides the input score characteristics and offers insights into the predictive model (community-based health insurance enrollment). Figure 4 displays the SHAP analysis summary graphic representation, which ranks features based on their importance in detecting the model (CBHI enrollment of reproductive-age women) by visualizing the SHAP values of each feature in the sample.

The feature ranking is determined by its position on the y-axis, which spans from high importance to low importance. The x-axis SHAP values for each attribute indicate its effect; positive SHAP values signify a positive association with the target class variable and vice versa. Additionally, a higher feature value is denoted by the color red, while a lower feature value is indicated by the color blue. The distribution is depicted by the overlapping jagged points. Consequently, the bar plot visualizes the top eight most important features, with “residence” occupying the top position.

The next dominant features include wealth, the age of the household head (age_hh_head), the husband’s educational level (husband_education_level), media exposure status (media_exposure), family size (family_size), and the sex of the household head (sex_hh_head). In contrast, the number of children under five (no_under_children) is less significant compared to the other features shown in the provided diagram. Consequently, residence, wealth, the age of the household head (age_hh_head), and family size (family_size) reduce the likelihood of community-based health insurance enrollment among reproductive-age women. Conversely, the husband’s educational level (husband_education_level), media exposure (media_exposure), the sex of the household head (sex_hh_head), and the number of children under 5 years of age (no_under5_children) increase the likelihood of CBHI enrollment. However, the number of children under 5 years of age (no_under5_children), media exposure (media_exposure), and the sex of the household head decrease the likelihood of CBHI enrollment among reproductive-age women in Ethiopia (Figure 4).

Additionally, a waterfall plot provides a detailed breakdown of how each feature contributes to the final prediction for a specific instance. Each bar in the plot represents a feature, with the length of the bar indicating the magnitude and direction of its contribution. *F*(*x*) represents the model’s predicted output or target value for the specific observation [0] analyzed in the waterfall plot. *E* [*f*(*x*) = 0.796] in the waterfall plot indicates the average or expected prediction of the model, serving as the starting point for understanding the influence of individual features on the final prediction *f*(*x*), without considering the effect of any feature contributions in the given sample. The baseline value is represented by a vertical dashed line in the middle.

Positive SHAP values, shown by bars to the right of the baseline, indicate features that have a positive effect on the prediction. Higher values of these features lead to increased predictions, while lower values correspond to decreased predictions. Conversely, negative SHAP values, represented by bars to the left of the baseline, indicate features that negatively impact the model’s prediction. Higher values of these features lead to lower predictions, while lower values result in higher predictions.

Furthermore, for a given observation, if the model’s output exceeds this value (*E* [*f*(*X*)] = 0.796), it corresponds to a positive class (enrolled in CBHI), while an output below this value corresponds to a negative class (not enrolled in CBHI). In addition, the waterfall plot helps clarify the importance and directionality of each feature’s impact on the model’s prediction, providing insights into how the model makes decisions for specific instances. Accordingly, for the first observation, the combination of positive contributions in red and negative contributions in blue shifts the expected value output to the final model output (*f*(*x*) = 0.818), which is classified as the positive class, i.e., enrolled in CBHI. As a result, residing in urban areas (0 = residence) and having a husband with primary education (1 = husband_educational_level) enhances the probability of enrolling in community-based health insurance.

**Figure 5:**
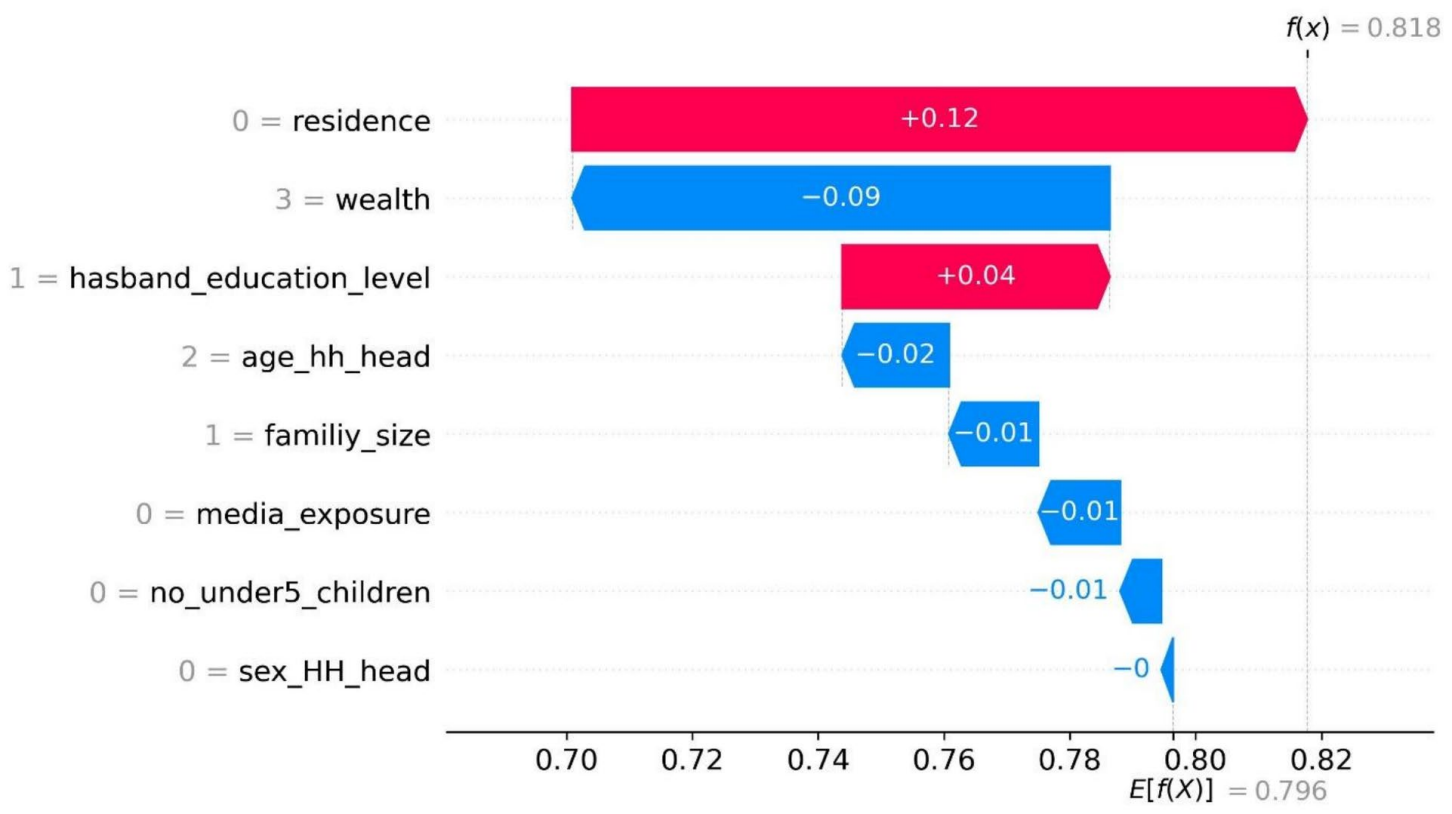
The waterplot display of CBHI enrollment (first observation) among reproductive-age women in Ethiopia, based on the EDHS 2019 dataset, 2024.

However, having greater wealth (3 = wealth), women aged 40 years and older (2 = age_hh_head), with a family size exceeding five members (1 = family_size), lacking media access (0 = media_exposure), women with two or fewer children under five (0 = no_under5_children), and where the head of the household is male (0 = sex_hh_head) decrease the likelihood of enrollment in community-based health insurance among reproductive-age women (Figure 5).

## Discussion

This study aimed to assess the importance of machine learning algorithms in predicting and identifying features that influence community-based health insurance enrollment among reproductive-age women in Ethiopia using the most recent EDHS 2019 dataset and SHAP analysis. Eight machine learning algorithms were employed for this purpose and trained with both imbalanced and balanced datasets using an 80/20 data split method. In this method, 80% of the dataset was used for model training, with tenfold cross-validation applied to the total observations, while the remaining 20% was reserved for model testing.

The performance of each machine learning algorithm was compared based on its accuracy and area under the ROC curve. Algorithms trained on a balanced dataset with the SMOTE oversampling technique demonstrate higher accuracy and ROC curve values than those trained on an imbalanced dataset.

As a result, the Random Forest model was found to be the most accurate, achieving an accuracy of 91.32% and an AUC of 0.885. Furthermore, it was optimized for its hyperparameters before conducting further analysis. Finally, the performance of the Random Forest machine learning algorithm after optimization reached 91.64% accuracy and 0.885 AUC.

A slight change in accuracy was observed. This finding was comparable to a study conducted in Ethiopia, which identified Random Forest as the best machine learning algorithm for predicting the unmet need for contraceptive use based on the PMA Ethiopia 2019 cross-sectional household and female survey, achieving an accuracy of 85% and an AUC of 0.93 (35). Additionally, this finding aligns with a study conducted in Ethiopia using the 2016 EDHS dataset to predict child vaccination among children aged 12–23 months (29). Furthermore, this finding is supported by a study done in East Africa using DHS data from 2011 to 2021 to predict delayed breastfeeding initiation among mothers with children under 2 months old, achieving an accuracy of 82% and an AUC of 0.84.

Regarding the second objective, SHAP analysis was employed to identify important features that influence the enrollment status of reproductive-age women in community-based health insurance. Consequently, residence and wealth status emerged as the primary predictors of community-based health insurance enrollment among reproductive-age women, followed by the age of the household head, the husband’s educational level/status, media exposure, family size, the sex of the household head, and the number of children under 5 years old, respectively. Additionally, SHAP analysis was used to understand the impact of each feature on community-based health insurance enrollment among reproductive-age women. Accordingly, living in urban areas positively affects community-based health insurance enrollment for reproductive-age women. These findings are consistent with a study conducted in Ethiopia using the EDHS dataset from 2019. This may be because women living in urban areas have greater access to information regarding the benefits of being a member of CBHI.

Furthermore, women living in urban areas had high economic capacity and could easily enroll in the program. Additionally, our study found that women whose husbands had primary education were more likely to enroll in community-based health insurance compared to those whose husbands were uneducated. This finding in-lined with a research conducted in Ethiopia and suggests that educated husbands possess greater knowledge about the advantages of program membership. Furthermore, educated husbands usually understand the impacts of out-of-pocket payments for accessing health services more clearly.

Furthermore, wealthier women are less likely to enroll in community-based health insurance. One possible explanation for this finding is that women with sufficient wealth may believe they can manage health catastrophes through direct out-of-pocket payments. This study contradicts previous research conducted in Ethiopia (31), Nigeria (32), and Bangladesh, which suggested that greater wealth is linked to higher asset losses when an unexpected health-related event occurs, prompting enrollment in program schemes. Another factor that influences CBHI enrollment is the age of the household head. Household heads aged 40 and older are less likely to enroll in community-based health insurance schemes compared to their younger counterparts. This finding aligns with previous studies conducted in Ethiopia based on demographic and health surveys (13, 27).

This may be due to older household heads having limited access to education, current information, and basic knowledge regarding the risks of out-of-pocket health payments. Additionally, this could be attributed to the differences in sociodemographic factors throughout the country. However, a study conducted in Nigeria (14) suggests that older age groups are more likely to enroll in CBHI schemes, as they face a higher risk of illness with increasing age. This may lead to greater investments in their health to protect themselves from financial uncertainties and health risks. Consequently, policymakers, initiatives, and program managers need to prioritize older age groups to promote their enrollment in these schemes.

Our study found that women with no media access were less likely to enroll in CBHI compared to those with media access. A possible explanation for this finding is that women without media access may perceive a higher risk of out-of-pocket payments for health services and medical emergencies, which could limit their willingness to enroll in CBHI. Furthermore, media coverage of the program could change community perceptions and encourage membership in the scheme. Therefore, the government should prioritize media coverage of the program.

Furthermore, our study found that women with more than five family members were less likely to enroll in community-based health insurance schemes. This finding is supported by a study conducted in Ethiopia (27). However, another finding suggests that women with larger family sizes were more likely to pay for CBHI schemes (31). This discrepancy may be attributed to the financial burdens faced by women with larger families when covering program premiums. Additionally, the study indicates that women with two or fewer children under five were less likely to enroll in CBHI compared to their counterparts. This could be due to the perception of these women’s ability to manage health costs through out-of-pocket payments. Finally, our study identified the sex of household heads as a potential predictor of CBHI enrollment among reproductive-age women in Ethiopia. Consequently, households with male heads were less likely to enroll in CBHI schemes than those headed by women. However, these results were not uniform across different study settings, possibly due to the sociodemographic characteristics of the participants. Therefore, policymakers need to address sex differences in household headship.

### Limitations and strengths of this study

The study’s key strengths include the recent EDHS and a large, nationally representative dataset. Furthermore, it employs advanced statistical analysis to uncover and pinpoint strong evidence essential for policymakers and program managers to make evidence-based decisions. Additionally, the SHAP analysis technique was employed to identify important features and visualize how each feature affects the model’s predictions. However, this study has certain limitations. The cross-sectional approach used to gather the EDHS data restricts our ability to draw causal conclusions regarding the examined variables. Since this investigation is a secondary analysis of EDHS data, several key variables were not included. Due to the retrospective nature of the survey based on questionnaires, recall bias may occur. The authors encourage future researchers to incorporate association rule mining techniques to identify the joint effect of each feature on the outcome variable.

## Conclusion

Machine learning algorithms and SHAP analysis were employed to predict and identify potential features influencing community-based health insurance enrollment among reproductive-age women. Among the machine learning models used, the Random Forest model was found to be the most effective for predicting community-based health insurance enrollment, achieving an accuracy of 91.64% and an area under the curve (AUC) of 0.885.

Additionally, SHAP analysis identified the key features influencing community-based health insurance enrollment among reproductive-age women. Specifically, urban residency and partners achieving primary education were significant factors that increased the likelihood of enrollment. Conversely, factors such as higher wealth, a household head aged 40 years of age and above, larger family size, lack of media access, having fewer children under 5 years of age, and a male household head were critical features that decreased the likelihood of CBHI enrollment.

Achieving the effects of these features helps tailor the program to meet its goal of addressing the financial difficulties faced by reproductive-age women. Furthermore, this approach offers valuable insights and identifies key patterns, enabling program managers and policymakers to make evidence-based decisions that enhance the enrollment of reproductive-age women in the program.

## Data Availability

The dataset for this study is publicly available on the Measure DHS program website (http://dhsprogram.com).

## Footnotes

^1^ http://dhsprogram.com

## Glossary

AUC: Area under curve
CBHI: Community-based health insurance
DT: Decision tree
EDHS: Ethiopian demographic and health survey
FN: False negative
FP: False positive
GNB: Gaussian Naive Bayes
KNN: K-nears neighbor
LBM: Light boost
LR: Logistic regression
ML: Machine learning
RF: Random forest
SDG: Sustainable development goal
SHAP: SHapley Additive exPlanations
SMOTE: Synthetic minority over-sampling technique
STATA: Statistical software for data science
SVM: Support vector machine
TP: True positive
TN: True negative
ROC: Receiver operating characteristic
AUC: Area under curve
UHC: Universal health care
XGB: XGBoost
